# Impaired glucose tolerance and indeterminate glycemia in cystic fibrosis

**DOI:** 10.1016/j.jcte.2021.100275

**Published:** 2021-11-16

**Authors:** Nader Kasim, Swapnil Khare, Zahre Sandouk, Christine Chan

**Affiliations:** a Division of Pediatric Endocrinology and Diabetes, Helen Devos Children’s Hospital, Grand Rapids, MI, USA; b Department of Endocrinology, Diabetes and Metabolism, Indiana University, Indianapolis, IN, USA; c Metabolism, Endocrinology and Nutrition Division, Internal Medicine Department, University of Michigan, Ann Arbor, MI, USA; d Department of Pediatrics, Division of Endocrinology, Children’s Hospital Colorado, University of Colorado Anschutz Medical Campus, Aurora, CO, USA

**Keywords:** OGTT, oral glucose tolerance, CF, cystic fibrosis, CFRD, cystic fibrosis related diabetes, AGT, abnormal glucose tolerance, INDET, indeterminate glycemia, CFTR, cystic fibrosis transmembrane conductance regulator, NGT, normal glucose tolerance, AUC, area under the curve, IVGTT, Intravenous glucose tolerance test, FEV1, Forced Expiratory Volume, FVC, Forced Vital Capacity, Oral glucose tolerance testing, Impaired glucose tolerance, Indeterminate glucose tolerance, Indeterminate glycemia, Abnormal glucose tolerance, Cystic fibrosis, Cystic fibrosis related diabetes

## Abstract

Oral glucose tolerance testing (OGTT) is the primary method to screen for and diagnose cystic fibrosis-related diabetes (CFRD). Diagnostic thresholds as currently defined are based on microvascular complications seen in type 2 diabetes. Abnormal glucose tolerance (AGT) refers to OGTT glucose elevations outside the normal range and encompasses both impaired and indeterminate glucose tolerance. Current guidelines define impaired glucose tolerance (IGT) as a 2-hour glucose of 140–199 mg/dL (7.8–11 mmol/L) and indeterminate glucose tolerance (INDET) as any mid-OGTT glucose ≥ 200 mg/dL (11.1 mmol/L) with a normal fasting and 2 h glucose. There is growing evidence that AGT also has associations with CF-centered outcomes including pulmonary decline, hospitalizations, and weight loss. Here we aim to review the historical emergence of glucose tolerance testing, review relevance to risk stratification for CFRD, discuss alternate cutoffs for identifying AGT earlier, and highlight the need for larger, future studies to inform our understanding of the implications of IGT and INDET on CF health.

## Highlights

• Oral glucose tolerance testing is used for screening, diagnosis, and risk stratification of cystic fibrosis related diabetes.
• Abnormal glucose tolerance in cystic fibrosis has prognostic utility with regards to progression towards overt diabetes, pulmonary function, weight loss, and mortality.
• Further research is needed to delineate the significance of impaired glucose tolerance and indeterminate glycemia within the CF population.
• Lower thresholds for indeterminate glycemia may be needed within the cystic fibrosis population.

## Introduction

Cystic fibrosis (CF) is a disease caused by autosomal recessive defects resulting in functional abnormalities of the cystic fibrosis transmembrane conductance regulator (CFTR) protein. Expression of the CFTR protein is ubiquitous and leads to multi-organ complications. The most common complication of CF after pulmonary decline is cystic fibrosis related diabetes (CFRD). CFRD has been strongly associated with lung function decline, poorer nutritional status, and increased mortality [1]. The natural history of CFRD has been well defined. Initially, there is defective first phase insulin response with eventual loss of second phase insulin secretion [2]. This decline in insulin secretion progresses with increasing age, resulting in a CFRD prevalence of 2% at age 10 years, 19% by adolescence, and 40% in early adulthood [3]. Given the high rates of diabetes in this population, oral glucose tolerance testing (OGTT) has a pivotal role in the screening and diagnosis of CFRD. Additionally, impaired glucose tolerance (IGT) and indeterminate glycemia (INDET) are categories of glucose tolerance that provide risk assessment for the progression of CFRD and its complications. In this review, we discuss the history of OGTT, evolution of glucose tolerance categories, and provide an overview of the literature to date focusing on IGT and INDET in the CF population.

## Historical emergence of glucose tolerance testing

The emergence of oral glucose tolerance testing stemmed from the realization that fasting blood glucose did not capture a significant portion of patients with type 2 diabetes. The emergence of glucose tolerance testing in the diagnosis of CFRD resulted from similar observations. IGT was formally described in the early 1950′s, in the context of pregnancy [4]. By 1960, glucose tolerance testing was ubiquitous but not standardized. Initially, multiple diagnostic thresholds and glucose doses were proposed [4], [5], [6], [7]. In 1964, the Medical and Scientific Section of the British Diabetic Association acknowledged that tolerance testing has a definitive role and highlighted the need for standardization. They proposed one of the first diagnostic classifications and thresholds for glucose tolerance testing and introduced the term pre-diabetes [5].

By the 1960′s, glucose tolerance testing was shown to be reproducible among patients with diabetes [6], and by the 1970′s, longitudinal data demonstrated retinopathy and correlations with IGT and progression to diabetes [8]. This led to the development of a consensus statement between the World Health Organization and the US- National Diabetes Data Group for the diagnosis of Type 2 diabetes in 1979. They recommended utilizing a 75 g glucose load for the OGTT and diagnostic thresholds for diabetes with a fasting glucose ≥ 140 mg/dL (7.8 mmol/L) and 2 h glucose of ≥ 200 mg/dL (11.1 mmol/L), and defined IGT as a 2 h glucose between 140 mg/dL (7.8 mmol/L) and 200 mg/dL (11.1 mmol/L) [9]. Guidelines continued to evolve and in 1997, the American Diabetes Association, followed by the World Health Organization, introduced the term impaired fasting glucose and lowered the fasting glucose diagnostic threshold for diabetes to 126 mg/dL (7 mmol/L) [10]. By the late 1990′s data continue to emerge showing increasing risk for microvascular disease with impaired and abnormal glucose tolerance [10].

Glucose tolerance testing in CF followed a similar historical path. As glucose tolerance testing became more ubiquitous, it was extended to diagnose other forms of diabetes. Glucose intolerance in CF was first described in 1969 with 42% of 31 patients demonstrating “CF of the pancreas”. This coincided with a diminished insulin response. Elevations in post-challenge glucose were observed as early as 1 h [11]. Glucose intolerance continued to be described in CF cohorts over time. However, the clinical uptake of OGTT faced challenges. These challenges included concerns about the relevance of diabetes in CF and inconvenience of testing. By the 1980’s and early 1990’s, as improvement in life expectancy in CF was recognized, the implications of CFRD and impaired glucose tolerance became more relevant [12]. It was also at this time that larger cohorts started to emerge due to increasing numbers of CF centers. This led to a better understanding of the natural history of glucose intolerance and CFRD. In 1991, Lanng and colleagues performed the first large cross-sectional study describing the prevalence of glucose intolerance in a Danish CF Center utilizing WHO criteria. Out of 210 individuals with CF, 15% had IGT and 7% had CFRD. The median age of IGT diagnosis was 18 years and the median age of CFRD diagnosis was 21 years [13]. With greater insight into the natural history and epidemiology of CFRD, it was recognized that diabetes was associated with increased CF-specific complications, including pulmonary function decline, weight loss, and increased mortality. This, in turn, led to the development of clinical care guidelines for the diagnosis and management of CFRD by the American Diabetes Association in 2010, with similar recommendations set forth by the European CF Society and International Society of Pediatric and Adolescent Diabetes [14], [15], [16].

## Description and classification of glucose tolerance

The oral glucose tolerance test (OGTT) is utilized for screening, risk stratification, and diagnosis of CFRD and guidelines recommend annual screening be performed starting at the age of 10 years [16], [17]. The standard OGTT is performed by checking a fasting plasma glucose, followed by an oral glucose load (1.75 g/kg, up to 75 g), and 2 h plasma glucose. Elevations in mid-OGTT glucoses have been well described in people with CF, prior to a diagnosis of CFRD, and guidelines recommend consideration be given to measuring mid-OGTT glucoses every 30 min during the 2-hour test [15]. In a patient who does not have clear symptoms of diabetes (polyuria, polydipsia, dehydration), testing should be repeated on a separate day during a period of stable health [16].

Glucose tolerance can be classified into five categories, which span the spectrum of euglycemia to overt diabetes (Table 1. These categories are: normal glucose tolerance (NGT), IGT, impaired fasting glucose (IFG), INDET, and CFRD. The term abnormal glucose tolerance encompasses all forms of glucose tolerance that are not NGT. Risk of clinical decline including pulmonary decline, poor lean body mass, increased hospitalizations, increased mortality, and microvascular disease, increases with worsening glucose tolerance.

**Table 1:** Glucose intolerance and associated CF-centered outcomes.

**INDET**				
	**Article**	**Year**	**Study design**	**Findings**
	Ode, K.L., et al.	2010	Retrospective matched-pair cohort study of children 6–9 years. OGTT performed from 1998 to 2003. Classified as NGT and AGT. No sub-analysis for INDET.	AGT increased risk of developing diabetes (OR 11). No difference in lung function or weight.
	Schmid, K., et al.	2013	Subsample of 521 patients from a prospective study who had 2 OGTTs performed between 2001 and 2009	IFT, IGT, and INDET predict CFRD.
	Sheikh, S., et al.	2015	80 pediatric subjects underwent OGTT. Cut off of 160 mg/dL for elevated 1 h glucose.	BG > 160 mg/dL predicted CFRD (OR 4). However, it was not associated with decreased lung function or weight loss.
	Nyirjesy, S.C., et al.	2018	Cross-sectional study on 42 non-diabetic CF patients who underwent MMTT and GPA testing.	Impaired insulin and c-peptide secretion at 1 h BG > 155 mg/dL. Further supported by abnormal proinsulin secretion with clamp studies.
	Piona, C., et al.	2021	232 CF patients underwent OGTT, mathematical modeling used to characterize 5 defined stages of glucose tolerance: NGT, AGT140, INDET, IGT, CFRD	AGT140 showed a differing mechanism than INDET and NGT
**IGT**				
	Lanng, S., et al	1992	Retrospective cohort of 38 CF patients, 6 years prior to diagnosis of CFRD. Matched controls.	IGT and CFRD associated with decrease in FEV1 and FVC. Also showed deterioration with NIH clinical score. No increase in lung infections. “Pre-diabetics” showed decrease in FEV1, FVC, BMI and weight
	Milla, C.E.	2000	Prospective study of 152 CF patients over 4 years who underwent routine screening OGTT.	FEV1 and FVC decline over 4 years with correlation of IGT and CFRD FH negative patients. Low insulin levels were also associated with IGT and CFRD FH neg. BMI and weight did not show decline nor did they correlate with IGT or CFRD FH neg.
	Tofé, S., et al.	2005	Cross-sectional study on 50 CF patient who underwent OGTT and IVGTT	Negative linear correlation of 2 hr BG to FEV1, FVC, BMI and weight. Significantly worse in IGT and CFRD relative to patients with NGT. Also showed increase in hospitalizations.
	Costa, M., et al.,	2007	Cross-sectional study on 114 non-diabetic CF patients matched with 14 controls who underwent OGTT	IGT and glucose AUC are associated with > 25% FEV1 reduction between upper and lower quartiles for AUC No association with IGT and weight

The clinical implications of INDET have been less well-defined, although it has also been associated with increased risk of development of diabetes.

## The relationship between glucose tolerance and beta cell dysfunction

The earliest defect in evolution of glucose intolerance is impairment of the first phase insulin response. With time there is continued loss of insulin secretion, thus impairing the second phase of insulin release [2]. Insulin resistance is not typically seen in early stages of dysglycemia in CF. In later stages of CFRD, insulin resistance is thought to be driven by age [18], increased frequency of intercurrent illnesses, and chronic inflammation. Chronic illness and inflammation also appear to exacerbate beta-cell dysfunction, and as a result, patients with CF have progressive worsening of glucose tolerance with time [19].

Given CFRD is primarily driven by impaired insulin secretion in its early stages, it would make sense to utilize glucose tolerance testing for CFRD screening. In one of the first longitudinal studies, 32 patients with CF with normal fasting glucose were assessed over 2 years for glucose intolerance and dysfunctional insulin secretion. Over this 2 year time span, glucose intolerance worsened from 37.5% to 50% despite having normal fasting glucose. Insulin secretion with glucose load also decreased [20]. In addition to oral glucose tolerance testing, these data were concurrently and later validated with intravenous glucose tolerance tests and clamp studies, leading to a more robust understanding of the first phase insulin secretion defects in CFRD. Clamp studies showed that beta cell dysfunction resulted predominantly from concomitant insulinopenia, with or without insulin resistance [21], [22]. In addition, insulin resistance has been further described in some patients with CFRD, especially with older age and acute illness.

A recent study identified unique defects in glucose handling (beta cell function, insulin clearance, and insulin sensitivity) by glucose tolerance stages in over 200 CF patients. Through mathematical modeling of OGTTs, the authors described progressive deterioration from NGT to AGT (intermediate glucoses > 140 mg/dL [7.8 mmol/L]) to IGT to INDET to CFRD. In addition to the known defects in insulin secretion described in CF, they determined that the loss of first phase insulin secretion is more pronounced in INDET relative to other non-diabetes glucose tolerance categories. They also described mild abnormalities of all three determinants of glucose regulation in individuals with mid-OGTT glucoses of 140–200 mg/dL (7.8 mmol/L) [23], a group not currently included in traditional OGTT classifications.

As the longitudinal progression of glucose intolerance with progression towards CFRD was validated overtime, oral glucose tolerance testing has served as the gold standard for CFRD screening, in the context of stable health. Although there are other methods to screen for diabetes, such as fasting glucose, random glucose, and hemoglobin A1c, they are less sensitive than the OGTT for detecting diabetes. There are other clinical situations in CF where hyperglycemia is more likely to occur and may be transient, such as initiation of tube feedings or acute illness. Screening for hyperglycemia in these circumstances is recommended with fasting and post-meal blood glucose checks [16].

## CF-specific outcomes associated with impaired glucose tolerance

The value of glucose tolerance testing continues to be of interest as clinical outcomes such as mortality, hospitalization, lung function, lean body mass loss, and weight loss are directly associated with a CFRD diagnosis. Increasingly, correlations between impaired glucose tolerance and indeterminate glucose tolerance with CF-relevant clinical outcomes are being described [13], [20], [24], [25], [26], [27].

### Lung function

In addition to overt CFRD, IGT has been associated with pulmonary decline. Longitudinal studies performed at CF centers in the late 1980′s and early 1990′s described significant deterioration in lung function with both impaired glucose tolerance and abnormal glucose tolerance occurring approximately 1 year before onset of CFRD [20], [28]. In a later paper, CFRD and IGT were negatively correlated with FVC and there was no significant difference between those who had IGT and CFRD in this cohort [27]. In another 4 year prospective study following 152 patients with CF, there was an approximately 10% decline of FEV1 and FVC over 4 years, with increased severity in the IGT and CFRD groups. Furthermore, insulin deficiency was associated with worse lung function [28]. In another cross-sectional observational study of 114 non-diabetic individuals who had CF, IGT along with glucose area under the curve (AUC) were directly associated with FEV1, showing over a 25% reduction between the lower and upper quartiles of AUC [29].

As the metabolic progression of diabetes is more of a spectrum, it is possible that milder dysglycemia may be contributing to pulmonary function decline. Proposed mechanisms include a progressive catabolic state driven by insulin insufficiency compounded deterioration of lean body mass, ultimately affecting cough and mucous clearing. In addition, glucose homeostasis in the airways has been shown to be altered in the presence of elevated blood glucose [19]. To highlight this, data have shown alterations in bacterial colonization with abnormal glucose tolerance, which in turn likely impacts exacerbations and overall clinical status [30], [31]. Similarly, elevations of glucose detected in alveolar secretions occur with blood glucose values as high as 144 mg/dL (8 mmol/L), a threshold in the realm of IGT and below the diagnostic threshold of CFRD [32]. This increase in glucose may provide nutritional substrate for bacterial growth, thus promoting infection, pulmonary exacerbation, and clinical deterioration. Further research to highlight mechanisms behind early impairments in glucose tolerance and pulmonary function decline are needed.

### Hospitalization and mortality

Worsened lung function leads to higher risk of hospitalization and mortality. In the last decade, data have shown direct associations between CFRD and mortality and clinical decline. In a large retrospective cohort study of over 8000 individuals with CF, hazard ratio for all-cause mortality was noted to be 1.3 (CI1.03–1.67) [24]. In a more recent retrospective study of 664 patients, it was shown that overall mortality has decreased over time with the improvements in screening and treatment of diabetes. Yet mortality was still noted to be 3.6 times higher in individuals with CFRD, especially when over 30 years of age, compared to those without diabetes [26]. These data provide indirect evidence that treatment of diabetes reduces mortality in CF. To date, there are no data to suggest a higher risk of mortality with impaired glucose tolerance.

Unlike mortality, risk of hospitalization has been strongly linked to impaired glucose tolerance. This risk was evident in the earlier studies outlining clinical decline years prior to CFRD diagnosis [20].

### Weight and lean body mass

In addition to pulmonary decline, the classic presentation of CF includes poor weight gain and low lean body mass, due to catabolism and pancreatic insufficiency. Insulin is an anabolic hormone and insulin deficiency, even prior to overt CFRD, may promote further catabolism. Longitudinal data from the last 2 decades have demonstrated reductions in weight and BMI up to 4 years before CFRD diagnosis that reverses with insulin treatment [20], [33]. In 2005, in a cross-sectional study of 50 patients with CF who underwent OGTT and IVGTT, those who had impaired glucose tolerance showed diminished insulin secretion with undernutrition [27].

## Indeterminate glycemia (INDET)

INDET is defined as a normal fasting glucose and normal 2 h post-challenge glucose with any intermediate OGTT glucose, ≥200 mg/dL (11.1 mmol/L) [16]. Abnormalities outside of the 2 h post-challenge glucose have long been described for all types of diabetes. However, its clinical significance is uncertain. The Diabetes Prevention Trial-1 first described the predictive nature of elevations in 1 h glucose in individuals at risk for Type 1 diabetes, [34] which has been incorporated into CFRD guidelines and demonstrated to also predict increased risk for CFRD.

In the mid 2000′s, larger studies in CF showed that elevated 30 min and 1 h glucose values were predictive of lung function along with other metrics such as glucose AUC [27], [29]. This was further augmented by insulin clamp studies showing beta cell dysfunction in individuals with INDET, studies characterizing beta cell dysfunction with glucose tolerance categories, and continuous glucose monitoring studies correlating CGM data to indeterminate glucose tolerance on OGTT [21], [23], [25], [35], [36].

### Outcomes associated with indeterminate glycemia

Like type 1 diabetes, the strongest association with INDET is progression to overt diabetes. In a retrospective matched-pair cohort study, children between the ages 6 to 9 years who either had IGT or INDET had a significantly higher chance of developing CFRD (3% versus 42%, OR 11) [37]. In a longitudinal prospective study of CF children and adults, IFG, IGT, and INDET were all associated with a higher risk of progression to CFRD (OR 2.81 for the subjects with INDET who progressed to CFRD) [38].

The utilization of a lower glycemic threshold for intermediate OGTT glucose values has been proposed as associations with CFRD progression have been shown at thresholds as low as 155 mg/dL (8.6 mmol/L), which is below the current definition for INDET [39], [40]. These data are further supported by demonstration of beta cell decline at these thresholds [23]. CGM data also provide supporting evidence of this [36], [41], [42], [43]. Recent studies by Prentice and colleagues have demonstrated that even in young children with CF, peak intermediate OGTT glucoses inversely correlate with weight and FEV1 decline, and CGM time > 140 mg/dL correlates with increased pseudomonas colonization and markers of pulmonary inflammation [35].

INDET has been associated with lower lung function, although data from different studies are conflicting. In a retrospective study of 101 individuals with CF, there was a strong negative correlation between 1 h glucose and FEV1 when corrected for BMI [44]. In a similar study of 252 patients, there was an approximately 10% reduction in FEV1 in the INDET subgroup relative to the NGT subgroup [45]. In 2018, a longitudinal evaluation of glycemic trajectories was performed, showing that lung function decline was not associated with INDET unless there was persistent worsening over multiple OGTTs [46]. This was also shown in another retrospective study of a French and Canadian cohort, showing that lung function was not associated with INDET [47].

Unlike IGT and AGT, abnormal weight loss has not been clearly associated with INDET [44], [45], [46]. Small reports, however, have suggested that treatment of early abnormalities with glargine may show modest improvement in BMI in those who inherently have a low BMI [48], [49].

Data on the impact of INDET on mortality, hospitalizations, and colonization are lacking.

## Treatment of indeterminate glucose tolerance and impaired glucose tolerance

There are limited data to suggest that treatment of INDET with insulin may improve CF-relevant clinical outcomes. In a subgroup of 25 individuals who were found to have INDET by OGTT, a 1 year trial of glargine was shown to reduce pulmonary function decline, although this study did not have a control group. Furthermore, direct comparison between the INDET subgroup and CFRD subgroup showed similar improvement in lung function and BMI. Overall FEV1 showed a 9% increase and the number of lung infections was reduced by approximately 40% compared to the previous 12 months [49]. Like INDET, studies have suggested benefit of early treatment of IGT. In a study of 6 patients with IGT, there was a 4% increase in FEV1 after initiation of 0.3u/kg of glargine over an approximately 2 year time span and an approximately 0.5 kg/m2 improvement in BMI [48]. However, these patients were preselected and not randomized and there was no control group. Hameed and colleagues [50] demonstrated improvements in weight and lung function with once daily detemir in a small group of CF patients with pre-diabetes. In contrast, in the CFRD-Therapy Trial by Moran and colleagues [51], a randomized control trial of insulin, repaglinide and placebo, the group with IGT receiving insulin did not show improvements in BMI.

## Conclusion and future directions

Current thresholds for diagnosing NGT, IGT and CFRD were derived from populations at risk for type 2 diabetes. In CF, these threholds reflect progressive deterioration of beta-cell function and are associated with clinical decline in CF. Studies to date assessing the benefits of insulin intervention for IGT and/or INDET are limited and inconclusive. A multi-center randomized controlled trial of insulin therapy for early glucose abnormalities is currently underway (CF - Insulin Deficiency, Early Action, ClinicalTrials.gov Identifier: NCT01100892).

More recent data suggests more aggressive thresholds for CF may identify those at risk for progression to overt CFRD and decline of CF centered outcomes such as lung function and weight. In addition, with the associations being described with INDET, it is possible that diagnostic thresholds may need to be altered to accommodate other time points in glucose tolerance testing, such as 30 min and 1 h glucose values.

The emergence of modulator therapy may also alter the progression of glucose intolerance and prevalence of CFRD with time. Prospective studies are needed to understand the relationship between glucose tolerance testing and prediction of clinical outcomes in a new era of highly effective modulator therapy, particularly if modulator therapy continues to mitigate the mortality, hospitalization, pulmonary, and nutritional outcomes in this population.

## Funding

The authors received grant funding from the Cystic Fibrosis Foundation as part of the EnVision CF: Emerging Leaders in CF Endocrinology II Program (Award number: KASIM19GE0).

### CRediT authorship contribution statement

**Nader Kasim:** Conceptualization, Writing – original draft, Writing – review & editing, Visualization. **Swapnil Khare:** Conceptualization, Writing – review & editing. **Zahre Sandouk:** Conceptualization, Writing – review & editing. **Christine Chan:** Conceptualization, Writing – review & editing, Supervision.
